# Snakebite-induced severe varicella: Exploring venom-related immune dysregulation in a case report

**DOI:** 10.1016/j.jdcr.2025.11.047

**Published:** 2025-12-11

**Authors:** Yu Wang, Yuemeng Wu, Ying Ma

**Affiliations:** Department of Dermatology, Huashan Hospital, Fudan University, Shanghai, P. R. China

**Keywords:** immune dysregulation, snakebite, varicella

## Introduction

Varicella, caused by the varicella-zoster virus (VZV), is usually self-limiting in immunocompetent individuals but can worsen under certain conditions, including immunosuppression or concurrent infections.^1^ Snakebites can lead to immune dysregulation, with venom components triggering inflammatory responses that may temporarily suppress the immune system, potentially increasing susceptibility to infections like varicella.^2^ This case reports a previously healthy 25-year-old man who developed severe varicella after a snakebite, suggesting that venom-induced immune alterations might contribute to the severity of varicella in otherwise healthy individuals.

## Case report

**Fig 1:**
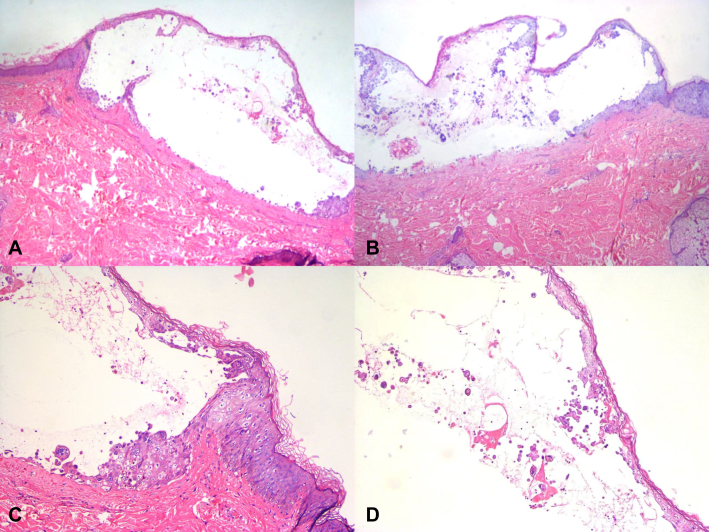
Histopathological image. **A** and **B,** Epidermal acantholysis with intraepidermal vesicle formation. **C,** Keratinocytes exhibit marked ballooning degeneration. **D,** Infiltration of multinucleated giant cells, intranuclear inclusion bodies, lymphocytes, plasma cells, and histiocytes in the blister fluid (hematoxylin and eosin, **A,B** - ×100; **C,D** - ×400).

A 25-year-old previously healthy man presented with a 5-day history of generalized rash and 1 day of fever. He had sustained a pit viper (Gloydius brevicaudus) snakebite on his left thumb 2 weeks earlier, for which he received antivenom therapy and supportive treatment. On day 0 of onset, the rash initially appeared on his left arm as erythematous vesicular lesions gradually spreading to the entire body, with pain but no pruritus. By day 5 after onset, he developed a widespread vesicular rash and started experiencing fever (maximum temperature 39.1 °C). Then he sought medical attention at a local hospital, where blood tests revealed thrombocytopenia (platelet count 34 × 10^3^/μL), severe liver dysfunction (alanine aminotransferase 1412 U/L, aspartate aminotransferase 1256 U/L), and electrolyte imbalances including hypokalemia (potassium, K 2.82 mmol/L), hyponatremia (sodium, Na 133.0 mmol/L), and hypocalcemia (calcium, Ca 1.88 mmol/L). A skin biopsy demonstrated epidermal acantholysis with intraepidermal vesicle formation, ballooning degeneration in keratinocytes, and infiltration of multinucleated giant cells, intranuclear inclusion bodies, lymphocytes, plasma cells, and histiocytes (Fig 1). Initial treatment included intravenous immunoglobulin (20 g/day), oral cetirizine, hepatoprotective therapy, and platelet support. However, symptoms worsened, prompting transfer to a tertiary hospital on day 7 after onset.

**Fig 2:**
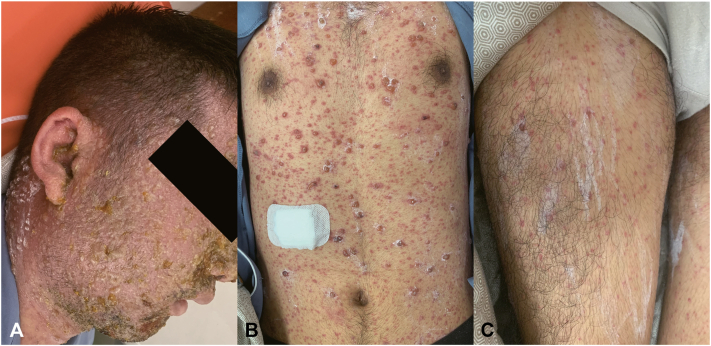
Clinical image. Widespread erythematous papules, vesicles, and hemorrhagic bullae with crusting on **(A)** head and face, **(B)** truck, and **(C)** limbs.

On admission, he had widespread erythematous papules, vesicles, and hemorrhagic bullae with crusting (Fig 2). Laboratory findings showed worsening thrombocytopenia (platelet count 7 × 10^3^/μL), elevated C-reactive protein (35.45 mg/L), coagulopathy (D-dimer >70.4 FEU mg/L), and persistent liver dysfunction (alanine aminotransferase 1163 U/L, aspartate aminotransferase 704 U/L). Immune-related laboratory tests revealed elevated pro-inflammatory cytokines (interleukin [IL]-6 15.7 pg/ml, IL-8 24.5 pg/ml) and an increased number of cytotoxic T lymphocytes (Tc 1396 cells/μL). Next-generation sequencing of vesicular fluid confirmed a high VZV sequence count (1,964,790 reads), along with low levels of *Streptococcus anginosus* and *Streptococcus intermedius*. A chest computed tomography scan was performed to exclude pulmonary involvement. Further history revealed the patient had no prior varicella infection and received varicella vaccination in childhood. He also denied exposure to immunosuppressive factors at home or work. The patient was diagnosed with severe varicella and treated with oral acyclovir (800 mg every 4 hours), intravenous methylprednisolone (60 mg/day), and intravenous immunoglobulin (25 g/day). Other treatment included low molecular heparin for thrombosis prevention, ceftriaxone for potential bacterial superinfection, hepatoprotective agents, and intensive skin care. After 2 weeks of treatment, his rash resolved and systemic abnormalities normalized.

## Discussion

Varicella is a highly contagious disease caused by the VZV, usually mild in children but potentially severe in adults, especially in immunocompromised individuals. Complications such as pneumonia, encephalitis, and coagulopathy are more frequently observed in these patients.^3^^,^^4^ Mechanistically, VZV establishes latency in sensory neurons, where restricted transcription maintains a poised state that can transition to lytic infection upon stress signals such as JNK/MAPK activation.^5^ Control of reactivation critically depends on T-cell–mediated immunity, particularly CD4^+^ and CD8^+^ responses primed by competent dendritic cells.^6^ Any condition that transiently impairs dendritic cell or T-cell function, or blunts antiviral stress responses, may tip the balance toward reactivation and severe disease. While established risk factors for severe varicella include immunosuppressive therapy, malignancy, and congenital immunodeficiencies, the immunomodulatory effects of snake envenomation remain underrecognized. Snake venom contains a wide range of bioactive components, including phospholipase A2, metalloproteinases, and serine proteases. These enzymes can trigger a systemic inflammatory response by promoting the release of pro-inflammatory cytokines such as tumor necrosis factor-α, IL-6, and IL-1β, potentially leading to cytokine storm and immune dysregulation.^7^ Additionally, venom has been shown to induce lymphocyte apoptosis and impair dendritic cell function, which may contribute to temporary immunosuppression.^2^ Venom-induced coagulopathy and endothelial injury can further compromise host defense mechanisms and vascular integrity.^8^ In this patient, who was previously healthy and had no known immunodeficiency, the development of severe varicella shortly after a snakebite raises the possibility of an association between venom-induced immune alterations and increased susceptibility to VZV reactivation or progression. The transient immunosuppressive state and excessive inflammatory storm response induced by the snake venom may have been important contributing factors to the severity of varicella in this patient. Previous reports have also described herpes labialis triggered by snakebite,^9^ possibly through similar venom-related immune dysregulation mechanisms. Furthermore, venom-induced coagulopathy and endothelial injury may have exacerbated the hemorrhagic nature of the varicella rash, contributing to the overall severity of the disease. Regarding treatment, the patient received prompt antiviral therapy and supportive care, including management of coagulopathy and hepatic dysfunction. In addition, systemic corticosteroid therapy was used, which is uncommon in the treatment of varicella. The use of corticosteroids in severe varicella remains controversial but may be considered in select cases with life-threatening systemic involvement,^1^^,^^10^ because corticosteroids can suppress excessive immune responses, such as cytokine storms.

In conclusion, snake venom may represent a potential risk factor for the development of severe varicella in otherwise healthy individuals. Further studies are needed to clarify the underlying mechanisms and clinical relevance.

## Conflicts of interest

None disclosed.
